# Effect of tocilizumab on intensive care patients with Covid-19 pneumonia, a retrospective cohort study

**DOI:** 10.3906/sag-2106-42

**Published:** 2021-11-09

**Authors:** Mehtap PEHLİVANLAR KÜÇÜK, Ahmet Oğuzhan KÜÇÜK, Ayşegül PEHLİVANLAR, Sevil AYAYDIN MÜRTEZAOĞLU, Kadir ÇOBAN, Gamze KILIÇ, Olcay AYÇİÇEK, Funda ÖZTUNA, Yılmaz BÜLBÜL, Tevfik ÖZLÜ

**Affiliations:** 1 Department of Chest Diseases, Division Of Intensive Care Medicine, Faculty of Medicine, Karadeniz Technical University, Trabzon, Turkey; 2 Department of Anesthesiology and Reanimation, Division of Intensive Care Medicine, Faculty of Medicine, Karadeniz Technical University, Trabzon, Turkey; 3 Department of Chest Diseases, Faculty of Medicine, Karadeniz Technical University, Trabzon, Turkey; 4 Department of Physical Medicine and Rehabilitation, Division of Rheumatology, Faculty of Medicine, Karadeniz Technical University, Trabzon, Turkey

**Keywords:** Antibodies, COVID-19, intensive care unit, mortality, SARS-COV-2, tocilizumab

## Abstract

**Backround/aim:**

In this study, the efficacy of an IL-6 antagonist, Tocilizumab, administered in the early period was studied in intensive care patients with COVID-19 pneumonia followed by hypoxic and systemic inflammation not receiving mechanical ventilation support.

**Materials and methods:**

Patients with COVID-19 pneumonia who have signs of hypoxia and systemic inflammation and/or who have acute bilateral infiltrates on chest radiograph and who received tocilizumab treatment were compared with the patients who received standard medical therapy. Patients who were followed up with COVID-19 pneumonia and respiratory failure between March 2020 and March 2021 were retrospectively evaluated in the study. A 400 mg – 800 mg iv dose (depending on weight) of Tocilizumab was administered. The primary endpoint was determined as intensive care unit mortality.

**Results:**

A total of 213 patients who were admitted with respiratory failure associated with COVID-19 to our third-level intensive care unit were evaluated. Of these patients, the study was conducted with 50 patients in the tocilizumab treatment group and 92 patients in the standard treatment group. During the intensive care period, 26 patients (28.3%) in the standard treatment group and 12 patients (24%) in the group receiving tocilizumab died. The adjusted hazard ratio for mortality in the tocilizumab group was 0.39 (95% confidence interval [CI], 0.186 to 0.808; p = 0.001 by log-rank test). During the intensive care period, 22 patients (24.8%) in the standart treatment group and 16 patients (32%) in the tocilizumab group were intubated. The adjusted hazard ratio for a primary outcome intubation in the tocilizumab group was 0.71 (95% confidence interval [CI], 0.355 to 1.424; p = 0.184 by log-rank test).

**Conclusion:**

Tocilizumab was administered with a low-dose steroid or following pulse steroid in intensive care patients followed up with COVID-19 pneumonia and evidence of hypoxia, and systemic inflammation was shown to reduce the intensive care all-cause mortality.

## 1. Introduction

Coronavirus disease 2019 (Covid-19) emerged in China in December 2019 and quickly led to a public health emergency^1^ [1]. The clinical spectrum of patients ranged from severe (14%) and critical (5%) cases of Covid-19 pneumonia to acute respiratory distress syndrome (ARDS) [2,3]. ARDS is the primary cause of poor prognosis in terms of mortality and morbidity in most of these patients. One potential consequence of COVID-19 is cytokine release syndrome (CRS), and its most severe form is secondary hemophagocytic lymphohistiocytosis. These syndromes are characterized by excessive inflammatory cytokine production. The cytokines most blamed for this cytokine storm are IL-6, IL-10, and tumor necrosis factor-a (TNF-a). Of these, IL-6 has been identified as a central player in toxicity due to its inflammatory properties through two signalling pathways: 1- the classical pathway and 2- the associated soluble receptor pathway. The onset of the pro-inflammatory response is determined by a significant increase in IL-6 levels. The plasma levels of IL-6 are also directly related to an increase of other inflammatory parameters in the blood, such as C-reactive protein (CRP), lactate dehydrogenase (LDH), and D-dimer [4].

Therefore, anti-cytokine therapies have been recommended as a treatment strategy in eligible COVID-19 patients [5–8]. Tocilizumab is a potential treatment option although its safety and efficacy in the COVID-19 patient population is still unclear. Tocilizumab is a US Food and Drug Administration approved IL-6 receptor antagonist widely used to treat CRS secondary to the chimeric antigen receptor T cell [9]. One of the advantages of tocilizumab is its long half-life, and its irreversible effect on IL-6 receptors in both the membrane-bound and soluble forms [10]. In the literature, studies using Tocilizumab in the treatment of COVID-19 have different clinical results [11–14]. According to the RECOVERY trial results, one of the most comprehensive of these studies, tocilizumab reduced the risk of death when given to hospitalized patients with severe COVID-19. The study also showed that tocilizumab shortened the time patients were successfully discharged from hospital and reduced the need for a mechanical ventilator. In this study, which included 177 centers and 35,000 patients in the UK, 2022 patients were included in the tocilizumab group and 2094 patients were included in the standard treatment group. Treatment with tocilizumab significantly reduced deaths: 596 (29%) of the patients in the tocilizumab group died within 28 days compared with 694 (33%) patients in the standard treatment group (rate ratio 0.86; [95% confidence interval (CI) 0 77 to 0.96]; p = 0.007), an absolute difference of 4%. This result is quite remarkable and promising.

The aim of this study was to report our experience with a series of COVID-19 patients treated in intensive care unit with tocilizumab. In this study, the effectiveness of tocilizumab treatment was evaluated in patients who were admitted to intensive care due to respiratory failure but did not need mechanical ventilation.

## 2. Materials and methods

### 2.1. Study group

The STROBE guideline was used as a guide for this manuscript. This study was carried out in Karadeniz Technical University Faculty of Medicine, a third-level, 16-bed Intensive Care Unit. The study was started after obtaining the approval of the local ethics committee (protocol number: 2021/24 date: 22.02.2021) and the Scientific Research Committee of the Ministry of Health with nº 2021-01-24T17_19_44. The study was also registered on the ClinicalTrials with protocol nº NCT04893031. In this study, COVID-19 pneumonia and respiratory failure cases admitted to our intensive care unit between March 2020 and March 2021 were retrospectively analyzed. The investigators designed the trial, collected the data, and performed the analysis. All cases were diagnosed based on typical radiological image using computed tomography (CT) or on the real time polymerase chain reaction (PCR) for Sars-CoV2 infection. The patients with severe and critical COVID-19 pneumonia were admitted to our intensive care unit. Severe illness, clinical signs of pneumonia (fever, cough, dyspnea, tachypnea), and one of the following: respiratory rate > 30 breaths/min, severe respiratory distress; or SpO2 < 90% on room air and, and presence of critical illness ARDS or respiratory failure requiring ventilation was defined as sepsis or septic shock [15]. These patients generally required high flow nasal cannula or invasive/noninvasive mechanical ventilation due to respiratory failure. The patients were followed until discharge from the intensive care unit or their death.

#### 2.1.1. Tocilizumab group

The patients with signs of hypoxia and systemic inflammation (SpO2 <90% on room air with an elevation of any two of the systemic inflammatory markers such as CRP, LDH and Ferritin) and/or acute onset of bilateral infiltrates on chest X-ray or rapid progression of existing infiltrates but who did not require mechanical ventilation at intensive care admission and were treated with tocilizumab.

#### 2.1.1. Standard treatment group

The patients who did not require mechanical ventilation in intensive care admission and who did not receive tocilizumab treatment at any period of hospitalization.

### 2.2. Exclusion criteria

**Figure 1:**
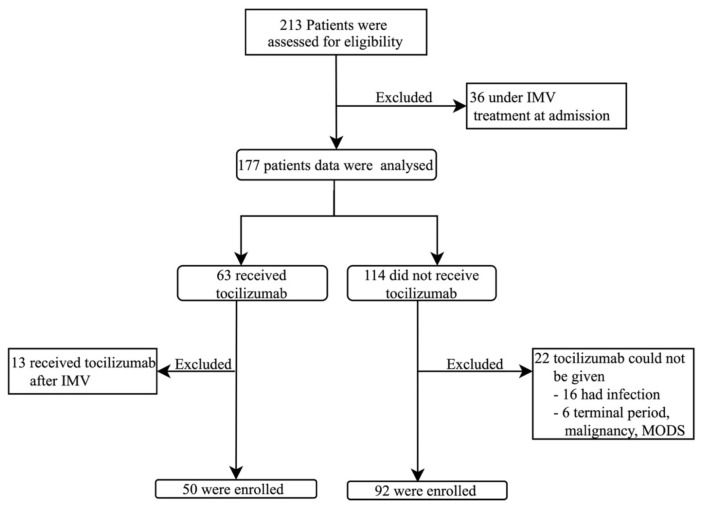
Study groups flow diagram.

The patients requiring mechanical ventilation during intensive care admission, patients <18 years of age, cases who received tocilizumab after being intubated during follow-up even though they were not intubated at admission, patients for whom tocilizumab is contraindicated^2^. The patients in the study group are shown in Figure 1.

### 2.3. Tocilizumab treatment approach

Treatment was initiated by detail explanation of the risks and benefits of tocilizumab to the patients or their first-degree relatives by obtaining approved consent. Signs of hypoxia and systemic inflammation (SpO2 <90% on room air and accompanying elevation of any two of the systemic inflammatory markers such as CRP, LDH and Ferritin) and/or acute bilateral infiltrations or rapid progression of existing infiltrations on chest radiography, without waiting to meet all of the Cytokine release syndrome (CRS) criteria, and with major treatment indications for tocilizumab treatment in our clinic.

Radiological follow-up was performed by daily bedside anteroposterior chest radiographs. The follow up inflammatory parameters are CRP, ferritin, and LDH levels. The increase in any two of these markers were considered significant for the inflammatory process. Since the IL-6 kit is not always available, it was not used in every patient. Tocilizumab dose was determined as 1 hour 8 mg/kg i.v. infusion (at a dose of 400–800 mg). A second dose was administered to some patients at 12–24 h intervals depending on the changes in their clinical condition.

The contraindications for tocilizumab are determined as increased procalcitonin levels with positive cultures (blood, urine, or sputum), indicating a suspected or confirmed bacterial infection, pregnancy, active tuberculosis, active hepatitis B-C, known allergy, and hypersensitivity^3^. Liver function tests and platelet counts were closely monitored during follow-up. All patients who received tocilizumab also received our standard treatment protocol which is; antiviral therapy (favipravir), low dose methylprednisolone (1–2 mg / kg) or pulse steroid (250 mg / day for 3 days) according to the clinical severity of disease, anticoagulant, vitamin C and vitamin D supplements. All patients who could adapt and benefit were followed in the prone/ semiprone position.

### 2.4. Follow-up parameters

Demographic characteristics of the patients, comorbidities, applied COVID-19 specific treatments, laboratory parameters at first admission, radiological evaluations, type of respiratory support given at the time of admission and during follow-up, on which day of the intensive care admission tocilizumab was given, arterial blood gas and PaO2/FiO2 values at the time of admission were recorded. The mortality and organ failure scores (Apache II and SOFA) of patients during ICU admissions, length of stay in intensive care unit, and patient outcomes were obtained from the medical records.

### 2.5. Study outcomes

The primary outcome of the study was the condition of all-cause mortality in tocilizumab and standard therapy patient groups. The secondary outcome was intubation in the tocilizumab or standard treatment group as assessed by an event-time analysis.

### 2.6. Statistical analysis

Data were analyzed with IBM SPSS V23 (Chicago, USA). Variables are examined by Shapiro – Wilk and Kolmogorov–Smirnov tests for the distributions. Continuous variables are expressed as mean ± standard deviation, or as median (interquartile range). The Student’s t test or the Mann–Whitney test was used to make comparisons. Categorical variables are presented as numbers (proportions) and were compared with the chi square test or the Fisher’s exact test where appropriate. Efficacy analyses were performed in the treat population with patients grouped according to treatment assignment. Analyses were adjusted according with APACHE II score. The primary outcome was estimated with the Kaplan – Meier method, and cumulative survive curves were compared between the groups with the stratified log-rank test. The stratified Cox proportional-hazards model was used to estimate the hazard ratio and 95% confidence interval. In this analysis, data on patients who survived and did not receive mechanical ventilation on or during intensive care unit stay were censored at the discharge from ICU. The primary and key secondary outcomes were evaluated in a hierarchical manner to control the overall trial-wide type I error rate at the 5% significance level.

## 3. Results

During the study period, 213 intensive care patients were evaluated in terms of eligibility for the study. Thirty-six patients were excluded from the study because they were supported by invasive mechanical ventilation at the time of admission to the intensive care unit. While 63 out of 177 patients received tocilizumab treatment, 114 patients did not receive tocilizumab treatment. In the tocilizumab group, 13 patients, who received treatment after being connected to an invasive mechanical ventilator, and 22 patients in the group who did not receive tocilizumab because of various reasons (16 patients with active infection, 6 patients with terminal disease, malignancy) were excluded from the study. Study analyzes were conducted with 50 patients receiving tocilizumab and 92 patients receiving only standard medical therapy (Figure 1).

The baseline characteristics of the patients are shown in Table 1. Ninety-five (66.9%) of the patients included in the population were male and 47 (33.1%) were female. The mean age was 62.94 ± 14.28 years. The youngest patient was 20 years old, and the oldest patient was 92 years old. Fifty-five of the patients (41.4%) had A rh (+) blood group and 39 (27.5%) of the patients were smokers, while 84 (54.2%) of them were alcohol consumers. While 104 of the patients (73.2%) had comorbidity, the most common comorbidity was hypertension in 82 (57.7%) patients. The baseline demographic and disease characteristics were generally balanced in the two groups. In the standard treatment (ST) and tocilizumab treatment (TT) groups, 22 patients (23.9%) and 8 patients (16%), respectively, used acetylsalicylic acid in their daily life, while 25 (27.2%) and 5 (10%) used ACE inhibitors, respectively. In the ST and TT groups, 19 patients (20.7%) and 6 patients (12%), respectively, had vaccinated Influenza vaccine, 12 patients (13%) and 2 patients (4%) had vaccinated Pneumococcal vaccine, respectively. The median concentration of laboratory values of groups was shown in Table 1. Serum median albumin level was statistically higher in the TT group with 32.6 g / L (IQR: 30.35–34.35) compared to the ST group 30 g / L (IQR: 27.95–33.6) (p = 0.030). The median scores of SOFA in the ST and TT groups were 3 (IQR: 2–4), 2.5 (IQR: 1–3.25), respectively (p = 0.207). The median scores of APACHE II in the ST and TT groups were 8 (IQR: 3.25–13) and 8 (IQR: 3.75–11.25), respectively (p = 0.774).

### 3.1. Primary and seconder endpoint

**Figure 2:**
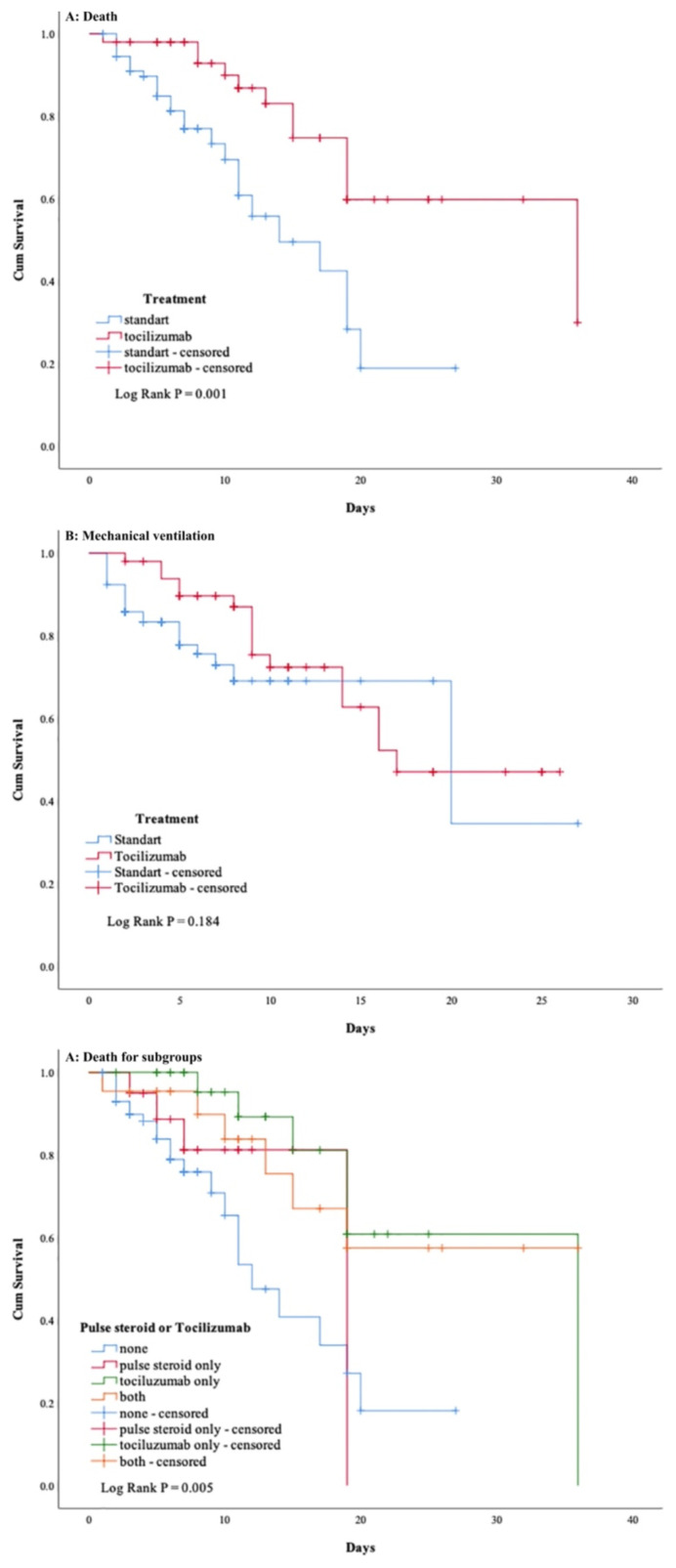
Kaplan–Meier analyses of efficacy outcomes. Shown are Kaplan–Meier curves for the time–to–event analyses of death (Panel A); time–to–event analyses of invasive mechanical ventilation (Panel B); and time–to–event analyses of death in subgroups as received tocilizumab, received pulse steroid, received both, received none (Panel C).

From the study patients, a total of 38 patients (26.7%) died in the intensive care unit (Table 2), 26 patients (28.3%) in the ST group and 12 patients (24%) in the TT group, respectively. The Kaplan–Meier curves for the time to death are shown in Figure 2A. The hazard ratio was estimated with adjustment for APACHE II score. The adjusted hazard ratio for a primary outcome mortality event in the tocilizumab treatment group was 0.39 (95% confidence interval [CI], 0.186 to 0.808; p = 0.001 by log-rank test). A total of 38 patients (26.7%) were intubated in the intensive care unit (Table 2), 22 patients (24.8%) were in the ST group and 16 patients (32%) were in the TT group. The Kaplan–Meier curves for the time to intubation are shown in Figure 2B. The hazard ratio was estimated with adjustment for APACHE II score. The adjusted hazard ratio for a primary outcome intubation event in the tocilizumab treatment group was 0.71 (95% confidence interval [CI], 0.355 to 1.424; p = 0.184 by log-rank test). Four groups as those who only take pulse steroids, those who only take tocilizumab, those who take both, and those who do not take both were created according to treatments. Mortality rates and occurrence time between subgroups were analyzed by Kaplan–Meier curves (Table 2 and Figure 2C). The hazard ratio was estimated with adjustment for APACHE II score. The group that received no treatment was considered as the base group and when the cox-regression analysis was compared with this base group the adjusted hazard ratio for a primary outcome mortality event in the pulse steroid treatment group was 1.13 (95% confidence interval [CI], 0.353 to 3.628), in the tocilizumab treatment group was 0.34 (95% confidence interval [CI], 0.132 to 0.854) and in the treatment with both group was 0.5 (95% confidence interval [CI], 0.185 to 1.355), p = 0.001 by log-rank test. Time dependent mortality rates were statistically and significantly lower only in the tocilizumab group compared to the other groups. In the regression analysis performed in the tocilizumab patient group, no significant relationship was found between the day of tocilizumab administration after hospitalization in the intensive care unit and mortality. [β=0.848 (C.I. %95; 0.562–1.279) p = 0.210]

### 3.2. Treatments and outcomes

The treatments given to the patients and the secondary outcome results are shown in Table 3 in a comparative manner according to the groups. Treatments performed in the ST group and TT group and their rates were as follows: favipiravir treatment 41 (44.6%) patients and 14 (28%) patients, respectively p = 0.079, daily 6 mg dexamethasone treatment for 14 (15.2%) patients and 6 (12%) patients respectively p = 0.784, low dose methylprednisolone treatment 53 (57.6%) patients and 33 (66%) patients, respectively p = 0.425, high dose methylprednisolone treatment 20 (21.7%) patients and 22 (44%) patients respectively p = 0.010, Vitamin D use 81 (88%) patients and 47 (94%) patients respectively p = 0.379, Vitamin C use in 82 (89.1%) patients and 46 (91%) patients respectively p = 0.770, Factor Xa inhibitor use in 75 (89.3%) patients and 44 (89.8%) patients respectively p = 1.000, Immune Plasma treatment 25 (27.5%) patients and 15 (30%) patients respectively p = 0.902, awake prone position method 26 (28.3%) patients and 33 (66%) patients respectively p < 0.001, vasopressor support in 27 (29.3%) patients and 16 (32%) patients respectively p = 0.891, sedation need in 40 (43.5%) patients and 35 (70%) patients respectively p = 0.007. There were statistically significant differences in treatments between the two groups in the use of high-dose steroids, the awake prone position, and the need for sedation (Table 3). There was a statistically significant difference on the day basis in the treatment durations (sedation, vasopressor, NIMV, IMV, HFNC) due to the longer median length of stay in the tocilizumab treatment group. In the secondary outcome targets, 21 (22.8%) patients in the ST group and 12 (24%) patients in the TT group developed acute kidney injury (p = 1.000). Hepatic failure rate was 16.3% with 15 patients in the ST group and 18% with 9 patients in the TT group (p = 0.982). The rates of discharge of the patients from ICU to services did not differ statistically between the ST group and the TT group (55 patients [59.8%], 37 patients [74%], p = 0.131, respectively).

## 4. Discussion

In our study, the clinical outcomes of tocilizumab treatment performed in patients with hypoxic COVID-19 pneumonia who do not receive mechanical ventilation support but have an indication for intensive care were evaluated. Study analyses were carried out after it was determined that the groups were standardized according to the APACHE II and SOFA scores. This study showed that the mortality rate of tocilizumab group (TT) was lower compared to the standard therapy group (ST). The results of our trial suggest that patients who are most likely to benefit from tocilizumab have moderate or severe disease (i.e. they have hypoxia but are not yet receiving mechanical ventilation) and that tocilizumab may add to the potential benefit of antiviral treatment and glucocorticoids. However, there was no decrease in the undergoing to mechanical ventilation in the tocilizumab treatment group. During intensive care period, 26 patients (28.3%) in the ST group and 12 patients (24%) in the TT group died. In our study, the distribution of patients who received antiviral and low-dose steroid therapy was balanced in both groups; however, the group that received tocilizumab also received a significantly higher rate (44%) of pulse steroids, which suggests that this group of patients were treatment-resistant and more severe.

While negative results related to tocilizumab treatment have been published on tocilizumab treatment at the beginning of the pandemic process, the current data and guidelines contain positive results on treatment efficacy of tocilizumab [12,16]. Literature data, including numerous studies such as COVACTA, EMPACTA, BACC By TOCI, CORIMUNO − TOCI, RCT − TCZ − COVID-19, revealed that tocilizumab does not provide a survival advantage [11,13,17–19]. While the COVACTA study was a randomized placebo-controlled study that enrolled a group of patients from moderate hypoxia to invasive mechanical ventilation, EMPACTA was similar to our study which included patients who did not need a mechanical ventilator; however, this study also showed no difference in the incidence of death from any cause, although it reduced the likelihood of progressing to mechanical ventilation or death compared to placebo and standard treatment [11,17]. The efficacy end point of survival without invasive or noninvasive mechanical ventilation by day 14 was achieved in the CORIMUNO-TOCI-1 trial but without a mortality benefit at day 28 [18]. More recently, the RECOVERY study shows that the benefits of tocilizumab extend to a broad group of patients receiving oxygen, with or without other forms of respiratory support, and that those benefits include a reduction in the need for invasive mechanical ventilation. RECOVERY study gives important results among these studies in terms of being multi-centered, randomized controlled, open-label and wide participation with 4116 patients. In this study, tocilizumab was administered to the patients on any oxygen support with a rapid increase in oxygen demand and a CRP level of ≥75 mg / l [12].

COVID-19 patients have a wide range of physiological and biological diversity [20]; therefore, despite the negative consequences, the succession of tocilizumab studies has not ceased due to the visible and rapid clinical improvements observed in some of the patient groups that have not yet been clearly identified. Our study is of importance because it has one of the first data about tocilizumab results in patients with COVID-19 pneumonia who need intensive care in Turkey. In our study, the effect of tocilizumab treatment in patients especially who do not need mechanical ventilation was investigated, and it was observed that all-cause mortality in intensive care reduced by 0.39 (CI 95%: 0.186–0.808). Although the tocilizumab treatment patient group was equivalent to the standard treatment group in terms of APACHE II and SOFA scores, inflammatory markers such as Ferritin, CRP, LDH were higher, and the first arrival P/F ratio (median 79.5) was statistically significantly lower in the tocilizumab treatment group. On the other hand, the decrease in mortality and the inability to prevent mechanical ventilation in the tocilizumab treatment group shows the successful results of mechanical ventilation, although intubation is needed in these patients. In the study, while all the tocilizumab treatment patients received low-dose methylprednisolone treatment, 19.7% received only tocilizumab without pulse steroid therapy, and 14% received tocilizumab treatment with pulse steroid. Only mortality of the tocilizumab treatment group was found to be significantly lower than the others (0.34 (CI 95%: 0.132–0.854)).

In the RECOVERY study, increased CRP was taken into account as it correlates with IL-6 as an inflammatory marker at follow-up [12,21]. Since IL-6 is not always a workable parameter in our laboratory in the decision of tocilizumab, we took as a base the level of markers such as ferritin, CRP, LDH (without determining a certain cut-off and the elevation of any two markers is sufficient), rapid increase in the patient’s O_2_ need and bilateral worsening of infiltrations on chest radiography. Therefore, we performed tocilizumab treatment in the early stages of clinical progression.

The importance of this study is given as follows. Since it is carried out in a single centered intensive care unit where a single responsible person (pulmonology and intensive care specialist) follows and arranges treatments for 24 h (consultation was not requested from any department for a treatment recommendation), there was no problem in standardization. The limitations of our study were that the study was a retrospective design and blood gas, and oxygenation values were not recorded in patients just before and after tocilizumab administration. In addition, radiological improvement after tocilizumab administration was not evaluated. Another limitation is that undesirable outcomes at the patients such as secondary infections were not evaluated.

In conclusion, tocilizumab reduces all cause mortality in intensive care when used with low-dose steroid or after pulse steroid in COVID-19 pneumonia patients with varying degrees of hypoxia and evidence of systemic inflammation. This benefit was found especially in patients who were given tocilizumab in the early period with clinical-radiological progression and increased oxygen demand. We recommend that new studies with a larger number of patients be planned prospectively in standardized groups.

## Figures and Tables

**Table 1:** Baseline Characteristics of the Patients.*

Characteristic	Standart Treatment (ST) (N = 92)	Tocilizumab Treatmnet (TT) (N = 50)	Total (N = 142)	p Value
**Age, mean ± std dev − yr**	**66.24 ± 12.48**	**56.86 ± 15.46**	**62.94 ± 14.28**	**<0.001**
**Male sex** − **no. (%)**	58 (63.04)	37 (74)	95 (66.9)	0.255
**A rh+ blood type** − **no. (%)**	37 (42)	18 (40)	55 (41.4)	0.968
**Height, mean ± SD − cm**	**168.5 ± 8.07**	**171.69 ± 9.45**	**169.61 ± 8.68**	**0.048**
**Weight, mean ± SD − kg**	82.75 ± 12.37	85.67 ± 13.13	83.77 ± 12.67	0.193
**Smoking** − **no. (%)**	26 (28.3)	13 (26)	39 (27.5)	0.927
**Cigarette, median (IQR) − pocket/yr**	**37.5 (30 — 50)**	**30 (16.25 – 40)**	**32.5 (30 − 48.75)**	**0.029**
**Alcohol using** − **no. (%)**	**61 (66.3)**	**23 (46)**	**84 (59.2)**	**0.019**
**Comorbidity** − **no. (%)**				
**Present of any comorbidity**	**73 (79.3)**	**31 (62)**	**104 (73.2)**	**0.042**
**Present of cardiovascular comorbidity**	61 (66.3)	24 (48)	85 (59.9)	0.052
**Hypertension**	59 (64.1)	23 (46)	82 (57.7)	0.056
**Present of respiratory comorbidity**	20 (21.7)	7 (14)	27 (19)	0.369
**Drugs or vaccine** − **no. (%)**				
**Acetylsalicylic acid**	22 (23.9)	8 (16)	30 (21.1)	0.374
**ACE inhibitors**	**25 (27.2)**	**5 (10)**	**30 (21.1)**	**0.029**
**Influenza vaccine**	19 (20.7)	6 (12)	25 (17.6)	0.288
**Pneumococcal vaccine**	12 (13)	2 (4)	14 (9.9)	0.138
**Median laboratory values (IQR)** †				
**C−reactive protein level − mg/L**	105.3 (46 − 168.7)	139.4 (68.6 − 182.5)	116.15 (61.5 − 176.6)	0.067
**White blood cell count − cells/mm** **^3^**	10160 (6862.5 − 13217.5)	12630 (7855 − 15660)	10710 (7015 − 14040)	0.080
**Neutrophil ratio − %**	90.2 (84.68 − 93)	90.9 (87.2 − 93.45)	90.4 (85.75 − 93.2)	0.230
**Lymphocyte ratio − %**	6.3 (3.93 − 9.45)	5.2 (3.45 − 8.45)	5.9 (3.7 − 9.1)	0.212
**Neutrophil to lymphocyte ratio**	14.91 (9.19 − 26.14)	17.6 (10.09 − 26.37)	15.24 (9.5 − 26.22)	0.359
**Platelet count − 10** **^3^** ** cells/mm** **^3^**	223 (166.25 − 294)	237 (165.5 − 318.5)	233 (166 − 297.5)	0.550
**Platelet to lymphocyte ratio**	413.45 (246.43 − 599.5)	392.1 (263.9 − 582.83)	413 (254.5 − 586.08)	0.950
**Albumine level − g/L**	**30 (27.95 − 33.6)**	**32.6 (30.35 − 34.35)**	**31.3 (29.1 − 34)**	**0.030**
**Procalcitonin level − μg/L**	0.17 (0.08 − 0.63)	0.15 (0.12 − 0.25)	0.17 (0.1 − 0.41)	0.505
**Blood urea nitrogen level − mg/dL**	**25 (18 − 37)**	**21 (14.75 − 27)**	**23 (17 − 34)**	**0.006**
**Creatinine level − mg/dL**	0.92 (0.75 − 1.2)	0.85 (0.69 − 1.07)	0.9 (0.71 − 1.12)	0.184
**Lactate dehydrogenase level − U**/L	456 (359 − 590)	522 (417 − 712)	477 (380 − 618.25)	0.024
**D−dimer level − mg/L**	1.2 (0.68 − 3.53)	0.94 (0.54 − 1.83)	1.01 (0.62 − 2.65)	0.097
**Troponin level − ng/L**	**11.65 (6.03 − 47.19)**	**7.13 (4.48 − 11.67)**	**9.84 (5.09 − 28.39)**	**0.007**
**Lactate level − mg/dL**	16 (12 − 25.75)	16.5 (12.75 − 24)	16 (12 − 24.25)	0.599
**Ferritin level − μg/L**	**604.5 (273.15 − 1055.5)**	**931 (516.3 − 1240.2)**	**680 (349.55 − 1114.75)**	**0.022**
**Median arterial blood gas values on day 1 under invasive mechanical ventilation (IQR)** ‡				
**pH value**	7.44 (7.41 − 7.49)	7.45 (7.4 − 7.48)	7.44 (7.41 − 7.49)	0.901
**FiO** **_2_** ** support − %**	80 (60 − 83)	80 (60 − 92.5)	80 (60 − 85)	0.072
**P/F ratio**	**87.8 (68.15 − 131.25)**	**79.5 (57.75 − 98.5)**	**85.5 (68 − 122.25)**	**0.040**
**PO** **_2_** ** value − mmHg**	65.7 (55.5 − 75)	60.2 (52.98 − 69.33)	63.2 (55.05 − 74.2)	0.139
**pCO** **_2_** ** value − mmHg**	35.3 (31.5 − 39.6)	37.85 (34.73 − 43.48)	36.8 (33.05 − 40)	0.062
**HCO** **_3_** ** value − mmol/L**	24.8 (23.2 − 27.4)	26.65 (25.08 − 27.75)	25.5 (23.5 − 27.6)	0.051
**sO** **_2_** ** value − %**	93 (88 − 95.5)	90.95 (86.53 − 94.23)	92 (87.9 − 95.15)	0.070
**Median score points (IQR)**				
**SOFA**	3 (2 − 4)	2.5 (1 − 3.25)	3 (1 − 4)	0.207
**APACHE II**	8 (3.25 − 13)	8 (3.75 − 11.25)	8 (3.75 − 13)	0.774

* Percentages may not total 100 because of rounding. IQR denotes interquartile range, FiO_2_ fraction of inspired oxygen, The P/F ratio equals the arterial pO_2_ divided by the FiO_2_, PO_2_ partial pressure of oxygen, pCO_2_ partial pressure of carbon dioxide, sO_2_ arterial oxygen saturation, SOFA sequential organ failure assessment, and APACHE II acute physiology and chronic health evaluation II.

† C−reactive protein levels were missing for 4 patients (3 in the ST group and 1 in the TT group), Albumine levels were missing for 7 patients (6 in the ST group and 1 in the TT group), Procalcitonin levels were missing for 16 patients (13 in the ST group and 1 in the TT group), Lactate dehydrogenase levels were missing for 8 patients (5 in the ST group and 3 in the TT group), D−dimer levels were missing for 12 patients (7 in the ST group and 5 in the TT group), Troponin levels were missing for 41 patients (25 in the ST group and 16 in the TT group), Ferritin levels were missing for 29 patients (22 in the ST group and 7 in the TT group).

‡ 22 patients were intubated in the ST group and 16 patients were intubated in the TT group.

**Table 2:** Time−to−Event outcomes in the treat population.*

Primary outcome	No. of Patients with event within ICU Stay (%)	Median No. of days to event (95% CI) †	Adj. Hazard Ratio (95% CI) ‡	Log−Rank P Value ¶
**Mortality**				
**Standart Treatment (N = 92)**	26 (28.3)	14 (6.643 **−** 21.357)	0.39 (0.186 **−** 0.808)	0.001
**Tocilizumab Treatment (N = 50)**	12 (24)	36 (11.781 **−** 60.219)		
**Invaziv mechanical ventilation**				
**Standart Treatment (N = 92)**	22 (24.8)	20 (3.222 **−** 36.778)	0.71 (0.355 **−** 1.424)	0.184
**Tocilizumab Treatment (N = 50)**	16 (32)	17 (NE **−** NE)		
**Mortality Subgroup** §				
**Standart Treatment (N = 72)**	22 (30.6)	12 (8.552 **−** 15.448)		0.005
**Pulse steroid Treatment (N = 20)**	4 (20.0)	19 (NE − NE)	1.13 (0.353 **−** 3.628)	
**Tocilizumab Treatment (N = 28)**	6 (21.4)	36 (NE − NE)	0.34 (0.132 **−** 0.854)	
**Both Treatment (N = 22)**	6 (27.3)	NE (NE − NE)	0.5 (0.185 **−** 1.355)	

* The treat population included the 142 patients (50 in the tocilizumab group and 92 in the not received tocilizumab group) who underwent study and received either tocilizumab or not before intubation or death. ICU denotes intensive care unit, Adj adjusted, and NE could not be estimated.

† The median time to an outcome event was estimated with the Kaplan–Meier approach.

‡ The adjusted Cox proportional−hazards model with APACHE II as an adjusteded factor was used to estimate the hazard ratio and 95% confidence interval. For APACHE II score 95% CI was 1.1 (1.045−1.156) in mortality outcome. For APACHE II score 95% CI was 1.11 (1.057−1.165) in invasive mechanical ventilatory outcome. For APACHE II score 95% CI was 1.1 (1.045−1.167) in mortality outcome between subgroups.

¶ The P value was calculated with the log−rank test. Significance testing was performed hierarchically to control the trial−wide type I error rate at a 5% significance level.

§ Each group was compared with the Standart Treatment group in the cox regression analyses.

**Table 3:** Comparison treatments and secondary outcomes of patients.*

Characteristic	Standart Treatment (ST) (N = 92)	Tocilizumab Treatmnet (TT) (N = 50)	Total (N = 142)	p Value
**Treatments** − **no. (%)**				
**Favipravir**	41 (44.6)	14 (28)	55 (38.7)	0.079
**Dexametasone – 6mg daily**	14 (15.2)	6 (12)	20 (14.1)	0.784
**Low dose metilprednisolone – < 1mg/kg/day**	53 (57.6)	33 (66)	86 (60.6)	0.425
**High dose metilprednisolone – > 250mg/day**	**20 (21.7)**	**22 (44)**	**42 (29.6)**	**0.010**
**D vitamin**	81 (88)	47 (94)	128 (90.1)	0.379
**C vitamin**	82 (89.1)	46 (92)	128 (90.1)	0.770
**Factor Xa inhibitors**	75 (89.3)	44 (89.8)	119 (89.5)	1.000
**Immunplasma**	25 (27.5)	15 (30)	40 (28.4)	0.902
**Prone position − awake**	**26 (28.3)**	**33 (66)**	**59 (41.5)**	**<0.001**
**Vasopressor support**	27 (29.3)	16 (32)	43 (30.3)	0.891
**Sedation support**	**40 (43.5)**	**35 (70)**	**75 (52.8)**	**0.007**
**Median treatments (IQR)**				
**Immune plasma dose − unit**	1 (0 − 1)	0 (0 − 1)	1 (0 − 1)	0.515
**Sedation − day**	**0 (0 − 5)**	**5 (0 − 8)**	**2 (0 − 6)**	**<0.001**
**Sedation free − day**	**4 (2 − 6)**	**6 (3 − 11)**	**4 (2 − 7)**	**<0.001**
**Vasopressors − day**	0 (0 − 1)	0 (0 − 2.25)	0 (0 − 2)	0.613
**Vasopressor free − day**	**4 (3 − 7)**	**11 (7 − 17)**	**6 (4 − 11)**	**<0.001**
**Non−invasive ventilation − day**	0 (0 − 1)	0 (0 − 0.25)	0 (0 − 1)	0.918
**Non−invasive ventilation free −day**	**5 (3 − 7.75)**	**11 (7 − 17.25)**	**7 (4 − 11.25)**	**<0.001**
**Invasive mechenical ventilation − day**	**4.5 (3 − 7)**	**11 (6 − 16)**	**6 (4 − 11)**	**<0.001**
**HFNC − day**	**2 (0 − 4.75)**	**7 (3.75 − 14)**	**3.5 (0 − 7.25)**	**<0.001**
**Outcomes** − **no. (%)**				
**Development of AKI**	21 (22.8)	12 (24)	33 (23.2)	1.000
**Hepatic Failure**	15 (16.3)	9 (18)	24 (16.9)	0.982
**Discharge to wards**	55 (59.8)	37 (74)	92 (64.8)	0.131

* Percentages may not total 100 because of rounding.

IQR denotes interquartile range, HFNC high−flow nasal cannula, and AKI acute kidney injury.

## Footnotes

1 WHO. World Health Organization (2021). COVID-19 Public Health Emergency of International Concern (PHEIC) Global research and innovation forum [online]. Website https://www.who.int/publications/m/item/covid-19-public-health-emergency-of-international-concern-(pheic)-global-research-and-innovation-forum [accessed May 4, 2021].

2 T.C. Sağlık Bakanlığı, Bilimsel Danışma Kurulu Çalışması (2020). Antisitokin-Antiinflamatuar Tedaviler, Koagülopati Yönetimi: Turkey [online]. Website moz-extension://1e25cedd-d1b7-894e-bbbd-18c83968a692/enhanced-reader.html?openApp&pdf=https%3A%2F%2Fcovid19.saglik.gov.tr%2FEklenti%2F39296%2F0%2Fcovid-19rehberiantisitokin-antiinflamatuartedavilerkoagulopatiyonetimipdf.pdf [accessed May 4, 2021].

3 T.C. Sağlık Bakanlığı, Bilimsel Danışma Kurulu Çalışması (2020). Antisitokin-Antiinflamatuar Tedaviler, Koagülopati Yönetimi: Turkey [online]. Website moz-extension://1e25cedd-d1b7-894e-bbbd-18c83968a692/enhanced-reader.html?openApp&pdf=https%3A%2F%2Fcovid19.saglik.gov.tr%2FEklenti%2F39296%2F0%2Fcovid-19rehberiantisitokin-antiinflamatuartedavilerkoagulopatiyonetimipdf.pdf [accessed May 4, 2021].
